# Placental Ras Regulates Inflammation Associated with Maternal Obesity

**DOI:** 10.1155/2018/3645386

**Published:** 2018-10-08

**Authors:** Stella Liong, Gillian Barker, Martha Lappas

**Affiliations:** ^1^Obstetrics, Nutrition and Endocrinology Group, Department of Obstetrics and Gynaecology, University of Melbourne, Victoria, Australia; ^2^Mercy Perinatal Research Centre, Mercy Hospital for Women, Victoria, Australia

## Abstract

Heightened placental inflammation and dysfunction are commonly associated in pregnant obese women compared to their pregnant lean counterparts. The small GTPase superfamily members known as the rat sarcoma viral oncogene homolog (Ras) proteins, in particular, the K-Ras and H-Ras isoforms, have been implicated to regulate inflammation. The aims were to determine the placental Ras expression and activity with maternal obesity and its role in regulating placental inflammation. Human placenta was obtained at term Caesarean section from lean and obese pregnant women to determine the effect of maternal obesity on Ras protein expression and activity. To determine the effect of Ras on inflammation induced by bacterial endotoxin LPS and proinflammatory cytokines TNF-*α* or IL-1*β*, the chemical inhibitor lonafarnib (total Ras inhibitor) and siRNA (siKRAS and siHRAS) were used. Total Ras protein expression together with combined K-Ras and H-Ras activity was significantly increased in the placenta of obese pregnant women and when stimulated with LPS, IL-1*β*, or TNF-*α*. Lonafarnib significantly suppressed LPS-, IL-1*β*-, or TNF-*α*-induced IL-6, IL-8, MCP-1, and GRO-*α* expression and secretion in placental tissue. Primary trophoblast cells transfected with siKRAS or siHRAS demonstrated only K-Ras silencing significantly decreased IL-1*β*-, TNF-*α*-, or LPS-induced IL-6, IL-8, and MCP-1 expression and secretion. Furthermore, siKRAS significantly reduced downstream ERK-1/2 activation induced by LPS. In trophoblast cells, ERK-1/2 signalling is required for IL-6, IL-8, MCP-1, and GRO-*α* secretion. These studies implicate a role for K-Ras in regulating inflammation in human placenta. Suppressing overactive placental K-Ras function may prevent adverse fetal outcomes complicated by maternal obesity.

## 1. Introduction

Obesity rates have been steadily increasing for the past 20 years [1], with nearly half of all women at reproductive age are overweight or obese [2]. Maternal obesity is associated with an increased risk of developing gestational diabetes, preeclampsia, and thromboembolic complications [3–5]. Offspring from obese pregnancies are at increased risk of excessive fetal overgrowth and fetal adiposity. These infants also have an increased risk of developing metabolic disease, including obesity, diabetes, cardiovascular disease, and certain cancers [6–11]. Given the adverse effects on fetal development associated with maternal obesity, it is important to elucidate the mechanism and consequence of maternal obesity on placental function.

Pregnancy is considered to be an inflammatory process [12]. This is exacerbated by maternal obesity; proinflammatory cytokines (including IL-1*β*, IL-6, and TNF-*α*) are increased in maternal plasma [13–17] and the placenta [18, 19] of obese pregnant women. Increased macrophage accumulation in placentas from obese women can lead to upregulated proinflammatory cytokine and chemokine production [17, 20]. Endotoxemia is also increased in the plasma and adipose tissue of obese pregnant women [13], which can further propagate inflammation in the placenta [13]. This heightened state of placental inflammation can lead to placental dysfunction, including the development of insulin resistance, excessive amino acid, and free fatty acid delivery to the fetus or reduced mitochondrial respiration and ATP generation [20–22].

The rat sarcoma viral oncogene homolog (Ras) proteins are members of the small GTPase superfamily. The activation of Ras proteins is controlled by the binding of guanine nucleotides, GTP or GDP; GTP-bound forms of Ras proteins are active whereas the GDP-bound forms of Ras proteins are inactive [23]. There are three known human Ras protooncogenes H-Ras, N-Ras, and K-Ras which can activate the downstream RAF-MEK-ERK signalling pathways important for cell cycle regulation, apoptosis, and cell differentiation [24]. Although the three isoforms share a high degree of homology, there is evidence which suggests that each Ras isoform are functionally unique. For example, K-Ras is required for embryonic development in mice, whereas mice without functional H-Ras and N-Ras developed normally [25]. Moreover, although growth factors such as epidermal growth factor (EGF), IL-3, and colony-stimulating factor (CSF)-1 can induce H-Ras, N-Ras, and K-Ras activation, recent studies have shown that EGF, IL-3, and CSF-1 preferentially activated K-Ras over H-Ras or N-Ras [26]. Notably, K-Ras and H-Ras have been reported to promote inflammation. For example, K-Ras can induce IL-6 and IL-8 production in tumor cells [27–29]. In MCF-7 human breast tumor cells, TNF-*α* and IL-1*β* act cooperatively with H-Ras leading to exacerbated IL-8 expression [30]. TNF-*α* can also promote the expression of activated GTP-bound H-Ras [30]. Activated H-Ras signalling has been demonstrated to enhance adult T cell leukemia (ATL) cells binding to ICAM-1 [31].

To our knowledge, there have been no studies on the role of Ras in regulating placental inflammation. Thus, the aims of this study were to assess the effect of maternal obesity on human placental Ras expression and activity. Given that K-Ras and H-Ras have been implicated to regulate inflammation in nongestational tissues, we used the Ras inhibitor lonafarnib, which inhibits farnesyltransferases, an enzyme involved in the posttranslational modification and activation of K-Ras and N-Ras proteins and to a lesser extent H-Ras activation. To determine which Ras isoform regulates inflammation in the placenta, we performed siRNA specific knockdown of K-Ras and H-Ras in human primary trophoblast cells stimulated by the bacterial endotoxin LPS or by proinflammatory cytokines TNF or IL-1*β*.

## 2. Materials and Methods

### 2.1. Tissue Collection

Approval for this study was obtained from the Mercy Hospital for Women's Research and Ethics Committee and written informed consent was obtained from all participating subjects. Women were invited to provide samples on the day of admission for surgery. All tissues were obtained at the time of term Caesarean section in the absence of labour to ensure there were no effects of labour on Ras expression. Indications for Caesarean section included a repeat Caesarean section or breech presentation. Women fulfilling any of the following criteria were excluded; diabetes (either preexisting or gestational diabetes), vascular/renal complication, multiple gestations, asthma, smokers, preeclampsia, chorioamnionitis, placental abruption, acute fetal distress, and women with any other adverse underlying medical conditions.

The placenta was obtained and processed within 15 min of delivery. Placental lobules (cotyledons) were obtained from various random locations of the placenta; the basal plate and chorionic surface were removed from the cotyledon, and villous tissue was obtained from the middle cross-section. Placental tissue was bluntly dissected to remove visible connective tissue and calcium deposits. Tissues were washed extensively with PBS and (i) immediately snap frozen in liquid nitrogen and stored at −80°C for analysis of protein expression by Western blotting or for Ras activity as detailed below, (ii) fixed and paraffin embedded for immunohistochemical analysis, (iii) immediately used for explant studies, or (iv) for trophoblast cell studies as detailed below.

For Ras protein expression and immunohistochemistry (IHC) studies, the placenta was obtained from women who entered pregnancy lean (BMI between 18–<25 kg/m^2^; *n* = 6 patients) or obese (BMI ≥ 30 kg/m^2^; *n* = 6 patients). The relevant clinical details of the subjects in this cohort are detailed as previously described [32]. For Ras activity studies, the placenta was obtained from women who entered pregnancy lean or obese (*n* = 15 patients per group). The relevant clinical details of the subjects in this cohort are detailed in Table 1.

### 2.2. Placental Explants

To determine the effect of inflammation on Ras protein expression, fresh placenta was obtained from nonobese women (*n* = 6 patients), and tissue explants were performed immediately as previously described [33]. Briefly, the placenta was finely diced and placed in DMEM at 37°C in a humidified atmosphere of 8% O_2_ and 5% CO_2_ for 1 h. Tissues were blotted dry on sterile filter paper and transferred to 24-well tissue culture plates (50 mg/well). The explants were incubated in 1 ml DMEM containing 100 U/ml penicillin G and 100 *μ*g/ml streptomycin. Tissues were incubated in the absence or presence of 10 *μ*g/ml LPS (derived from *Escherichia coli* 026:B6; Sigma-Aldrich; St. Louis, MO, USA), 10 ng/ml TNF-*α* (PeproTech; Rocky Hill, NJ, USA), or 5 ng/ml IL-1*β* (PeproTech; Rocky Hill, NJ, USA) for 20 h. The concentration of LPS, TNF-*α*, and IL-1*β* used was based on our previous studies which produced a robust inflammatory response in human placental explants [32]. Tissues were stored at −80°C for analysis of Ras activity and protein expression by Western blotting as detailed below.

To determine the effect of the Ras on inflammation, placental tissue explants were performed using the farnesyltransferase inhibitor lonafarnib (*n* = 6 patients). For these explant experiments, tissues were incubated in the absence or presence of 50 *μ*M lonafarnib (AdooQ BioScience; Irvine, CA, USA) for 60 min before the addition of 10 *μ*g/ml LPS, 10 ng/ml TNF-*α*, or 5 ng/ml IL-1*β* for 20 h. After final incubation, tissue and media were collected separately and stored at −80°C for further analysis as detailed below. The concentration of lonafarnib was based on previous studies in nongestational tissues [34] and an initial dose-response (data not shown).

### 2.3. Primary Human Trophoblast Cell Culture

Isolation and purification of primary villous trophoblast cells were performed from fresh placenta from nonobese pregnant women. Placental villous cytotrophoblasts were isolated as previously described [35] by DNase/trypsin digestion and purified by separation on a Percoll gradient. Briefly, placental villous tissue (~25 g) was dissected and washed in saline and then digested three times in a HEPES-buffered salt solution containing 0.25% trypsin and 0.2 mg/ml DNAse. Tissue was shaken at 37°C for 30 min. The cytotrophoblast cells were separated on a Percoll gradient and resuspended in standard cell culture medium (5.5 mM glucose, 44.5% DMEM, 44.5% Ham's-F12, and 10% fetal calf serum supplemented with antibiotics). The cells were plated on 24-well plates at a density of 5 × 10^5^ cells per well. The cells were cultured at 37°C in 8% O_2_ and 5% CO_2_ atmosphere, and the cell culture media was changed daily.

For the RAS siRNA studies, transfection of trophoblast cells was performed as previously described [35] using K-Ras siRNA (siKRAS), H-Ras siRNA (siHRAS), and negative control siRNA (siCONT) obtained from Ambion (Thermo Fisher Scientific; Scoresby, Vic, Australia). Briefly, cells were transfected with 100 nM siCONT, siKRAS or siHRAS, and 0.3% Lipofectamine 2000 (Life Technologies, Mulgrave, Victoria, Australia), incubated for 24 h, and removed, and fresh medium was added to wells. After 66 h (total culture time), cells were treated with or without 1 *μ*g/ml LPS, 10 ng/ml TNF-*α*, or 1 ng/ml IL-1*β*, and the cells were incubated at 37°C for an additional 24 h. The concentration of LPS, TNF-*α*, and IL-1*β* used was based on our previous studies which produced a robust inflammatory response in primary human trophoblast cells [32]. Cells were collected and stored at −80°C until assayed for Western blotting or mRNA expression by qRT-PCR as detailed below. Media was collected and stored at −80°C until assayed for cytokine release as detailed below. Cell viability was assessed by the 3-(4,5-dimethyl-2-thiazolyl)-2,5-diphenyl-2H-tetrazolium bromide (MTT) proliferation assay as we have previously described [36]. Experiments were performed on cells isolated from the placenta obtained from 5 patients.

To determine the effect of the ERK inhibitor U0126 on proinflammatory cytokines, trophoblast cells (after 66 h culture time) were treated with 1 *μ*g/ml LPS, 10 ng/ml TNF-*α*, or 1 ng/ml IL-1*β* in the presence or absence of 5 *μ*M U0126 (ERK1/2 inhibitor), and the cells were incubated at 37°C for an additional 24 h. The concentration of U0126 used is based on previous studies in human myometrial cells [37] and an initial dose-response (data not shown). Media was collected and stored at −80°C until assayed for cytokine and chemokine release, as detailed below. Experiments were performed on cells isolated from the placenta obtained from 5 patients.

### 2.4. Ras Activity Assay

Assessment of Ras activity was performed using Ras GTPase ELISA Kit according to the manufacturer's instructions (Abcam, Cambridge, MA, USA). Briefly, 50 mg of placental tissue were homogenised in 500 *μ*l of complete lysis buffer provided by the kit. To measure Ras activity, 100 *μ*g of lysate were added into a 96-well plate coated with Raf-RBD proteins fused to GST. Bound Ras proteins were detected by Ras-specific antibodies, and Ras activity was quantified by chemiluminescence. The analysis was performed using Microplate Manager Software version 6 (Bio-Rad Laboratories, Hercules, CA, USA). The limit of detection of the assay was 0.6 *μ*g/ml.

### 2.5. RNA Extraction and Quantitative RT-PCR (qRT-PCR)

Total RNA was extracted from tissues using TRIsure reagent according to the manufacturer's instructions (Bioline, Alexandria, NSW, Australia), as previously described [35]. RNA concentration and purity were measured using a NanoDrop ND1000 spectrophotometer (Thermo Scientific, Pittsburgh, PA). RNA purity and concentration were determined via the A_260_/A_280_ ratio. RNA (0.5 *μ*g for tissues and 0.2 *μ*g for cells) was converted to cDNA using the high-capacity cDNA reverse transcription kit according to the manufacturer's instructions (Applied Biosystems; Waltham, MA, USA). The RT-PCR was performed using the CFX384 Real-Time PCR detection system (Bio-Rad Laboratories; Gladesville, NSW, Australia) using 100 nM of predesigned and validated QuantiTect primers (primer sequences not available) (Qiagen; Chadstone Centre, Vic, Australia). K-Ras and H-Ras primers used were specific to their own isoforms and did not cross-react with other Ras isoforms. Ct values of genes of interests were normalised against the averaged Ct values of two housekeeping genes (succinate dehydrogenase complex flavoprotein subunit A (SDHA) and tyrosine 3-monooxygenase/tryptophan 5-monooxygenase activation protein zeta (YWHAZ)). Of note, there was no effect of experimental treatment on SDHA or YWHAZ mRNA expression. Fold differences were determined using the comparative Ct method. The response to LPS, TNF-*α*, or IL-1*β* between patients varied greatly, as we have previously reported [38]. Thus, data are presented as fold change in expression relative to the expression level in the LPS, TNF-*α*, or IL-1*β* treatment groups, which was set at 1.

### 2.6. Western Blotting

Protein extraction and Western blotting were performed as previously described [39]. Twenty micrograms of protein were separated into 10% polyacrylamide gels and transferred to nitrocellulose. Blots were incubated in either 1 *μ*g/ml mouse monoclonal anti-Ras (clone RAS10; Merck Millipore; Billerica, MA, USA), 1 *μ*g/ml mouse monoclonal antiphosphorylated (Tyr204) ERK (p-ERK) (sc-7383; Santa Cruz Biotechnology; Santa Cruz, CA), or 1 *μ*g/ml total ERK1/2 (sc-93; Santa Cruz Biotechnology; Santa Cruz, CA, USA) prepared in blocking buffer (5% skim milk in TBS with 0.05% Tween-20) for 16 h at 4°C. Membranes were viewed and analysed using the ChemiDoc XRS system (Bio-Rad Laboratories; Gladesville, NSW, Australia). Semiquantitative analysis of the relative density of the bands in Western blots was performed using Quantity One 4.2.1 image analysis software (Bio-Rad Laboratories, Hercules, CA, USA). The levels of Ras were normalised to the levels of *β*-actin (Sigma, St. Louis, MO, USA). The levels of p-ERK were normalised to the levels of total ERK1/2.

### 2.7. Enzyme Immunoassays

Assessment of IL-6 and IL-8 cytokine release was performed using CytoSet™ sandwich ELISA according to the manufacturer's instructions (Life Technologies, Carlsbad, CA). The limit of detection of the IL-6 and IL-8 assays was 16 and 12 pg/ml, respectively. The release of MCP-1 and GRO-*α* was performed using DuoSet™ sandwich ELISA according to the manufacturer's instructions (R&D Systems Inc., Minneapolis, MN). The limit of detection of GRO-*α* and MCP-1 assay was 31 and 15 pg/ml, respectively. The inter- and intra-assay coefficients of variation (CV) for all assays were less than 10%.

### 2.8. Statistical Analysis

Statistics were performed on the normalised data unless otherwise specified. All statistical analyses were undertaken using GraphPad Prism (GraphPad Software, La Jolla, CA, USA). For two sample comparisons, either a paired or unpaired Student's *t*-test was used to assess statistical significance between normally distributed data; otherwise, the nonparametric Mann-Whitney U (unpaired) or the Wilcoxon (matched pairs) tests were used. For all other comparisons, the homogeneity of data was assessed by Bartlett's test, and when significant, the data were logarithmically transformed before further analysis using a repeated measure one-way ANOVA (with Fisher's LSD post-hoc testing to discriminate among the means). Statistical significance was ascribed to a *p* value < 0.05. Data were expressed as mean ± SEM. SEM values calculated from between-subject variance.

## 3. Results

### 3.1. Effect of Preexisting Maternal Obesity on Placental Ras Expression

**Figure 1:**
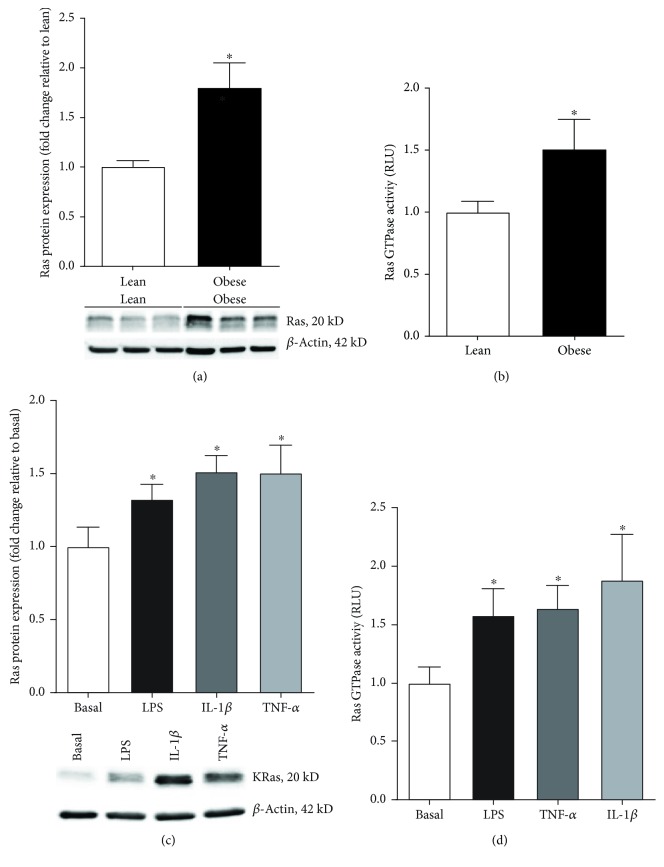
Placental Ras expression with maternal obesity and with exposure to inflammation. Human placenta was obtained from lean and obese women at the time of term Caesarean section. (a) Ras protein expression was assessed by Western blot and normalised to *β*-actin (*n* = 10 patients per group). The average densitometry of the Ras doublet band was determined. A representative Western blot from 6 patients is shown. (b) Ras GTPase activity was measured in the placenta of lean and obese women (*n* = 15 per group). (a, b) For all data, the fold change was calculated relative to the lean group. Data are displayed as mean ± SEM. ^∗^*p* < 0.05 vs. lean (Student's *t*-test). (c, d) Human placenta was incubated with 1 *μ*g/ml LPS, 10 ng/ml TNF-*α*, or 1 ng/ml IL-1*β* for 24 h (*n* = 6 patients per group). (c) Ras protein expression was analysed by Western blotting and normalised to *β*-actin. The fold change was calculated relative to basal. (d) Ras GTPase activity was measured in the placenta exposed to LPS, TNF-*α*, and IL-1*β*. (c, d) The fold change was calculated relative to basal. Data are displayed as mean ± SEM. ^∗^*p* < 0.05 vs. basal (paired Student's *t*-test). A representative Western blot from 1 patient is shown.

Western blotting was performed to characterise the expression of Ras in the placenta from lean and obese women (*n* = 6 patients per group). The Ras antibody used for Western blotting detects H-Ras (21.3 kD), K-Ras (21.7 kD), and N-Ras (21.2 kD) isoform. As shown in Figure 1(a), a doublet band at approximately 20–22 kD in size was detected by the Ras antibody in human placenta. Quantification analysis was performed using the average densitometry of both bands. Ras protein expression was significantly upregulated in the placenta obtained from obese pregnant women when compared to placenta obtained from lean pregnant women (Figure 1(a)). Several attempts to measure the protein expression of the specific isoforms of Ras (H-Ras, K-Ras, N-Ras) by Western blotting with commercially available antibodies were unsuccessful.

For the Ras activity assay, tissues were obtained from lean and obese pregnant women at term Caesarean section in the absence of labour (*n* = 15 patients per group). Demographic data of the participants are summarised in Table 1. The Ras activity assay detects both H-Ras and K-Ras isoform activity. As shown in Figure 1(b), Ras activation was significantly increased in the placenta of obese pregnant women compared to lean women.

### 3.2. Effect of Proinflammatory Mediators on K-Ras Expression

Obese pregnancies are characterised by endotoxemia [13] and inflammation [13–17]. Thus, the next aim was to determine if the bacterial product LPS and the proinflammatory cytokines TNF-*α* and IL-1*β* could affect Ras expression in human placenta. Treatment of human placental tissue with LPS, TNF-*α*, or IL-1*β* significantly increased Ras protein expression (Figure 1(c)) and activity (Figure 1(d)).

### 3.3. Effect of Ras Inhibitor Lonafarnib on Proinflammatory Cytokines in Human Placenta

**Figure 2:**
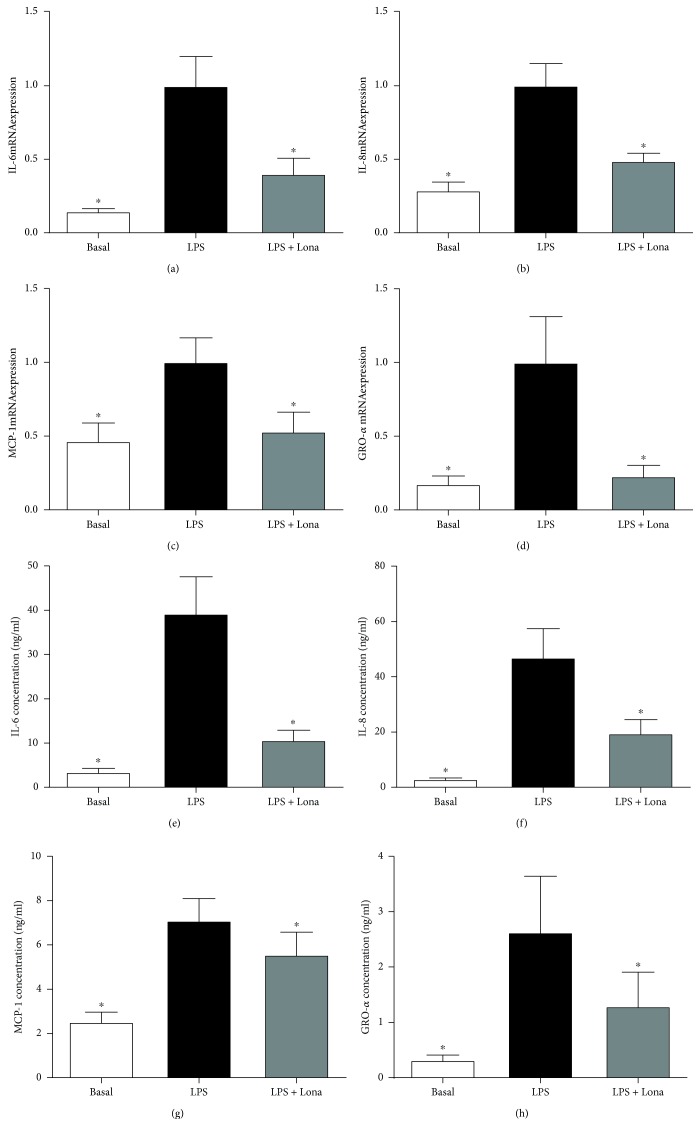
Effect of Ras inhibitor lonafarnib on LPS-induced inflammation in human placenta. Human placenta was incubated with 10 mg/ml LPS in the absence or presence of 50 *μ*M lonafarnib (lona) for 20 h (*n* = 6 patients). (a–d) IL-6, IL-8, MCP-1, and GRO-*α* mRNA expression was analysed by qRT-PCR, and the fold change was calculated relative to LPS. (e–h) The incubation medium was assayed for concentration of IL-6, IL-8, MCP-1, and GRO-*α* release by ELISA. All data are displayed as mean ± SEM. ^∗^*p* < 0.05 vs. LPS (repeated measures one-way ANOVA with Fisher's LSD post-hoc test).

**Figure 3:**
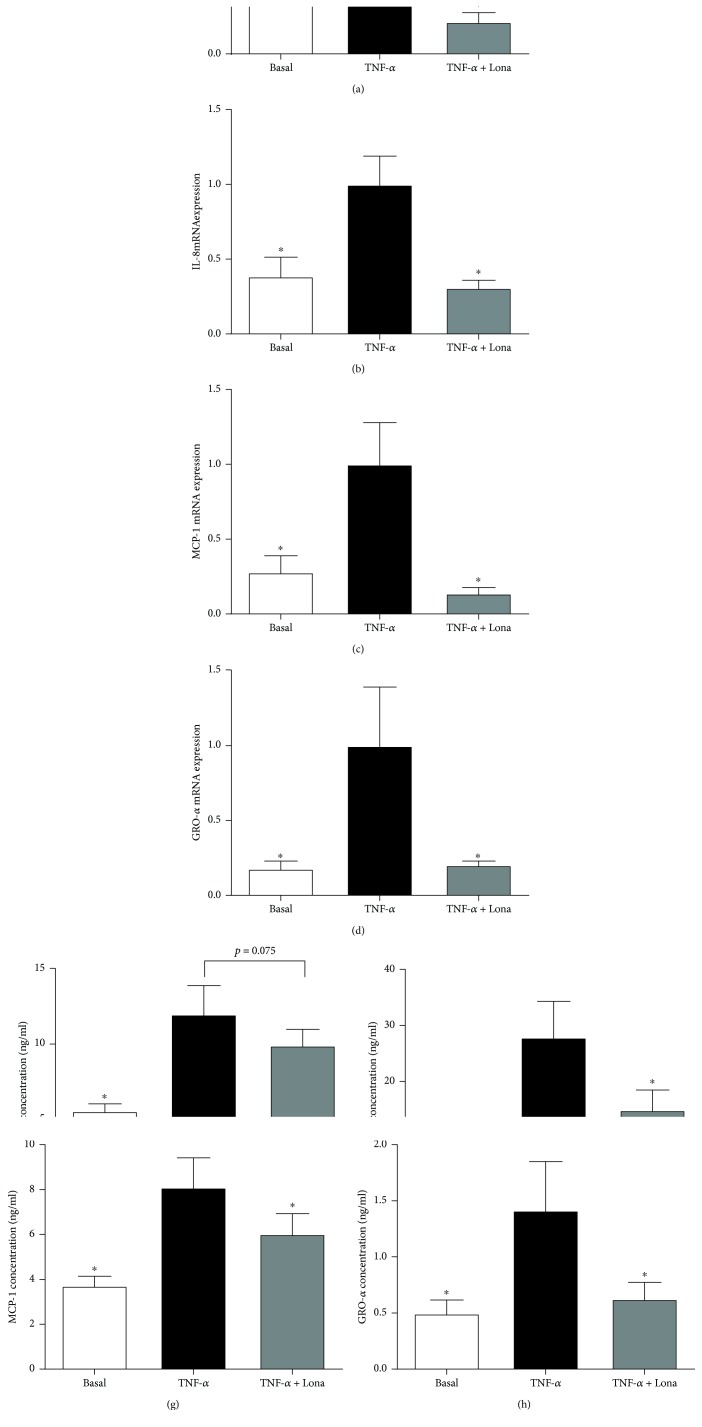
Effect of Ras inhibitor lonafarnib on TNF-*α*-induced inflammation in human placenta. Human placenta was incubated with 10 ng/ml TNF-*α* in the absence or presence of 50 *μ*M lonafarnib (lona) for 20 h (*n* = 6 patients). (a–d) IL-6, IL-8, MCP-1, and GRO-*α* mRNA expression was analysed by qRT-PCR, and the fold change was calculated relative to TNF-*α*. (e–h) The incubation medium was assayed for concentration of IL-6, IL-8, MCP-1, and GRO-*α* release by ELISA. All data are displayed as mean ± SEM. ^∗^*p* < 0.05 vs. TNF-*α* (repeated measures one-way ANOVA with Fisher's LSD post-hoc test).

**Figure 4:**
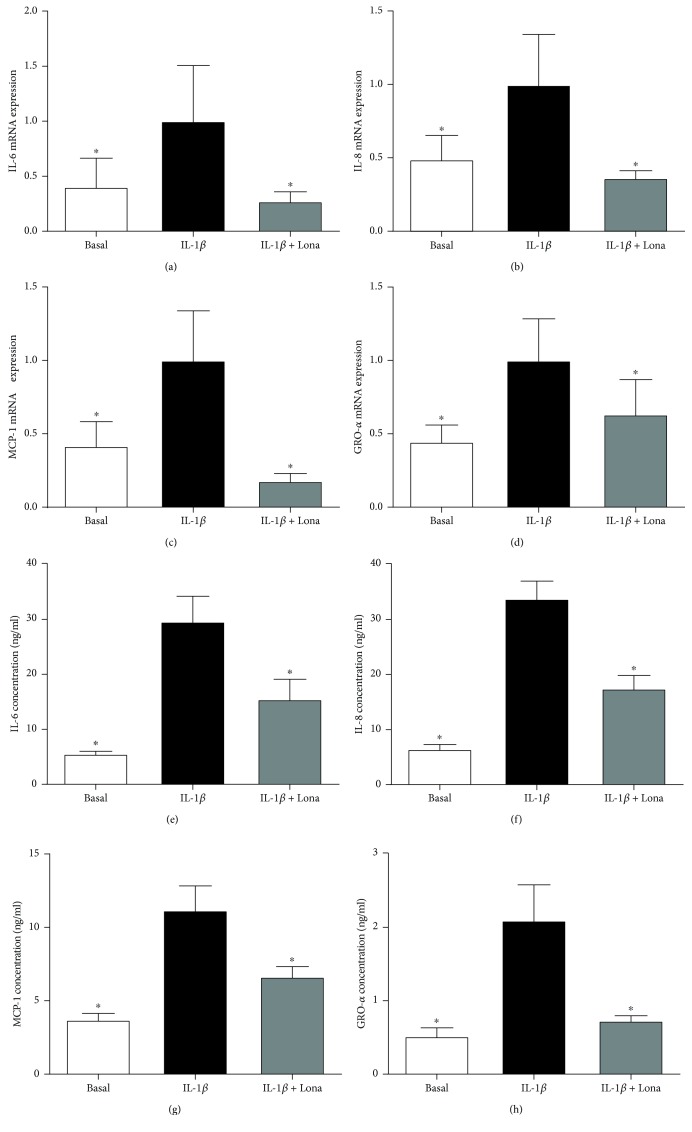
Effect of Ras inhibitor lonafarnib on IL-1*β*-induced inflammation in human placenta. Human placenta was incubated with 5 ng/ml IL-1*β* in the absence or presence of 50 *μ*M lonafarnib (lona) for 20 h (*n* = 6 patients). (a–d) IL-6, IL-8, MCP-1, and GRO-*α* mRNA expression was analysed by qRT-PCR, and the fold change was calculated relative to IL-1*β*. (e–h) The incubation medium was assayed for concentration of IL-6, IL-8, MCP-1, and GRO-*α* release by ELISA. All data are displayed as mean ± SEM. ^∗^*p* < 0.05 vs. IL-1*β* (repeated measures one-way ANOVA with Fisher's LSD post-hoc test).

Functional studies were next undertaken to investigate if Ras is involved in the production of proinflammatory cytokines induced by LPS, TNF-*α*, or IL-1*β*. To test this, we used the farnesyl transferase inhibitor lonafarnib, which inhibits the posttranslational modification and activation of the three Ras isoforms H-Ras, K-Ras, and N-Ras [40, 41]. The data for LPS, TNF-*α*, and IL-1*β* are presented in Figures 2, 3, and 4, respectively. As expected LPS, TNF-*α*, or IL-1*β* significantly increased IL-6, IL-8, MCP-1, and GRO-*α* mRNA expression and secretion. Pretreatment with lonafarnib significantly decreased the expression and secretion of IL-6, IL-8, MCP-1, and GRO-*α* induced by LPS and IL-1*β*. TNF-*α*-induced expression and secretion of IL-8, MCP-1, and GRO-*α* were also significantly reduced with lonafarnib treatment. Although lonafarnib significantly reduced TNF-*α*-induced expression of IL-6, there was however no significant reduction in TNF-*α*-induced secretion of IL-6 by lonafarnib.

### 3.4. Effect of siKRAS and siHRas on Proinflammatory Cytokines in Villous Trophoblast Cells

Studies in nongestational tissues have reported the K-Ras and H-Ras isoforms to regulate inflammation [42, 43]. In contrast, there is limited information on the role of N-Ras and inflammation. Thus, in our subsequent studies, we focused on the effect of K-Ras and H-Ras on placental inflammation. For these studies, we performed siRNA knockdown of K-Ras and H-Ras in primary human trophoblast cells. There was a 50% decrease in K-Ras and 79% decrease in H-Ras mRNA expression. A MTT cell viability assay showed no effect on cells transfected with siKRAS or siHRAS compared to siCONT (data not shown).

**Figure 5:**
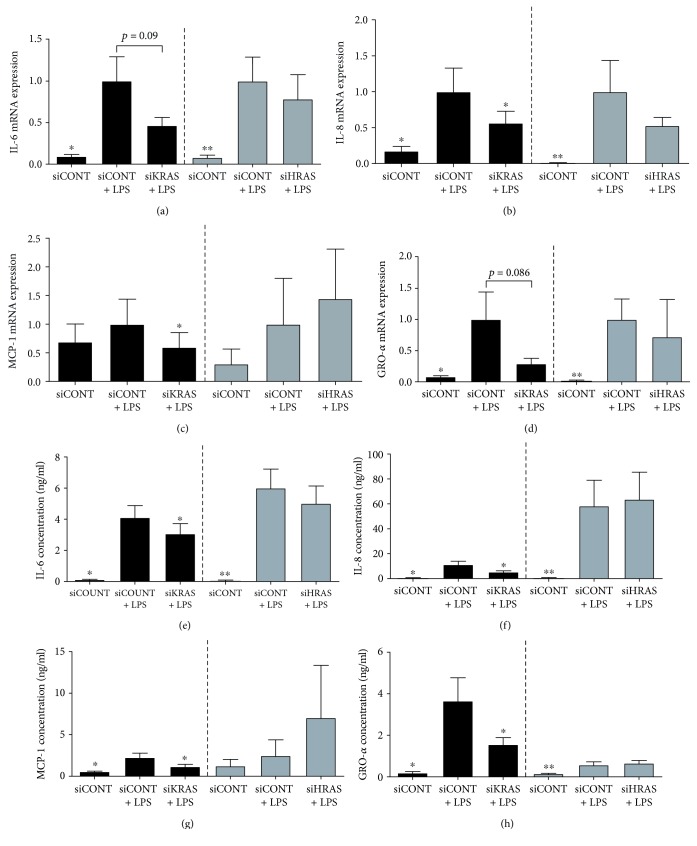
Effect of siKRAS and siHRAS on LPS-induced inflammation in human primary trophoblast cells. Human primary trophoblast cells were transfected with 50 nM siKRAS, 50 mM siHRAS, or 50 mM siCONT for 48 h then treated with 1 *μ*g/ml LPS for an additional 24 h (*n* = 5 patients). (a–d) IL-6, IL-8, MCP-1, and GRO-*α* mRNA expression was analysed by qRT-PCR, and the fold change was calculated relative to LPS-stimulated siCONT-transfected cells. (e–h) The incubation medium was assayed for concentration of IL-6, IL-8, MCP-1, and GRO-*α* release by ELISA. Data are displayed as mean ± SEM. ^∗^*p* < 0.05 vs. LPS-stimulated siCONT-transfected cells (repeated measures one-way ANOVA with Fisher's LSD post-hoc test).

**Figure 6:**
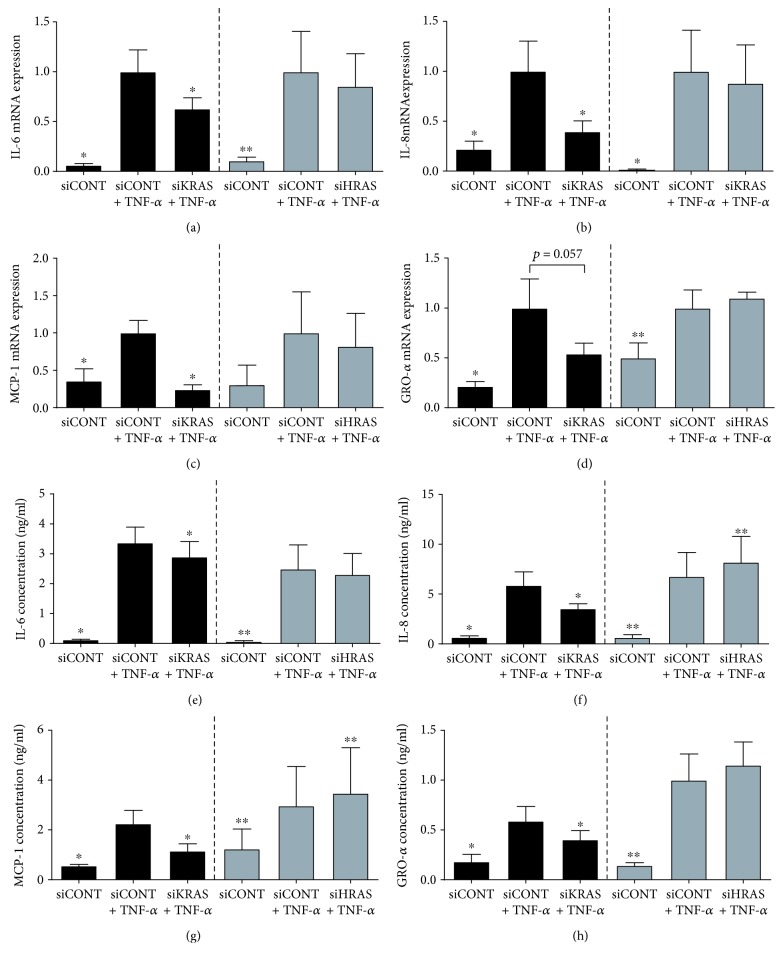
Effect of siKRAS and siHRAS on TNF-*α*-induced inflammation in human primary trophoblast cells. Human primary trophoblast cells were transfected with 50 nM siKRAS, 50 nM siHRAS, or 50 nM siCONT for 48 h then treated with 10 ng/ml TNF-*α* for an additional 24 h (*n* = 5 patients). (a–d) IL-6, IL-8, MCP-1, and GRO-*α* mRNA expression was analysed by qRT-PCR, and the fold change was calculated relative to TNF-*α*-stimulated siCONT-transfected cells. (e–h) The incubation medium was assayed for concentration of IL-6, IL-8, MCP-1, and GRO-*α* release by ELISA. Data are displayed as mean ± SEM. ^∗^*p* < 0.05 vs. TNF-*α*-stimulated siCONT-transfected cells (repeated measures one-way ANOVA with Fisher's LSD post-hoc test).

**Figure 7:**
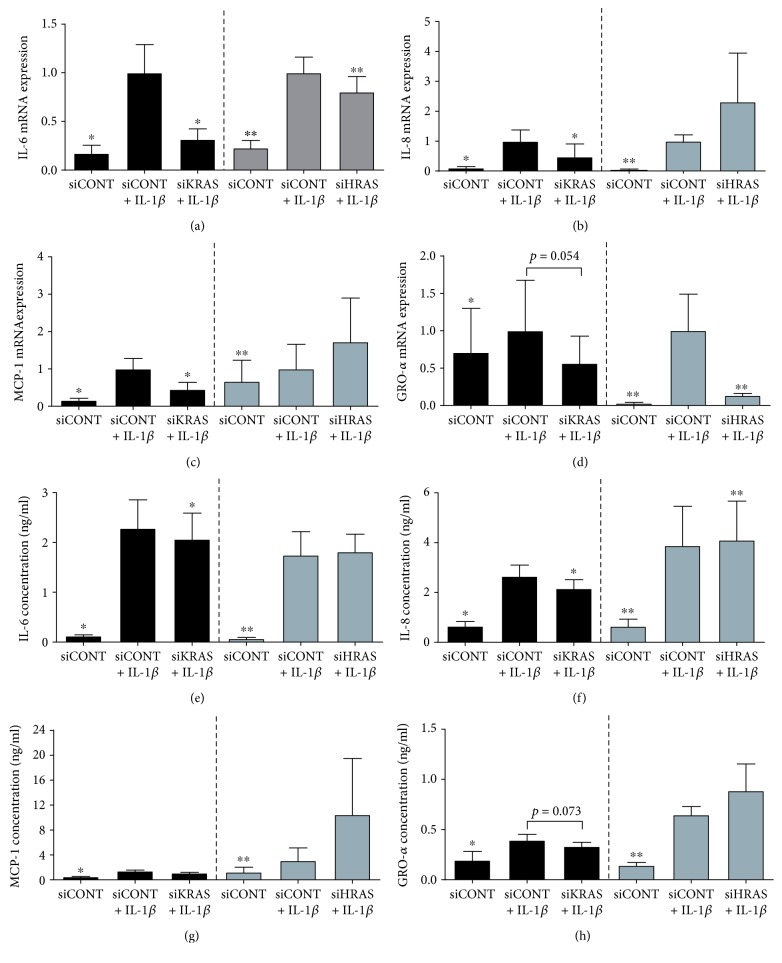
Effect of siKRAS and siHRAS on IL-1*β*-induced inflammation in human primary trophoblast cells. Human primary trophoblast cells were transfected with 50 nM siKRAS, 50 nM siHRAS, or 50 nM siCONT for 48 h then treated with 1 ng/ml IL-1*β* for an additional 24 h (*n* = 5 patients). (a–d) IL-6, IL-8, MCP-1, and GRO-*α* mRNA expression was analysed by qRT-PCR, and the fold change was calculated relative to IL-1*β*-stimulated siCONT-transfected cells. (e–h) The incubation medium was assayed for concentration of IL-6, IL-8, MCP-1, and GRO-*α* release by ELISA. Data are displayed as mean ± SEM. ^∗^*p* < 0.05 vs. IL-1*β*-stimulated siCONT-transfected cells (repeated measures one-way ANOVA with Fisher's LSD post-hoc test).

For subsequent experiments, after siRNA transfection, cells were treated with LPS (Figure 5), TNF-*α* (Figure 6), or IL-1*β* (Figure 7). As expected, LPS, TNF-*α*, or IL-1*β* significantly increased IL-6, IL-8, MCP-1, and GRO-*α* mRNA expression and secretion in siCONT-transfected cells. In cells transfected with siKRAS, although there was a significant decrease in LPS-induced IL-6 and GRO-*α* secretion, the reduction in IL-6 and GRO-*α* mRNA expression did not reach statistical significance (*p* = 0.09 and *p* = 0.086, respectively) (Figures 5(a), 5(e), 5(d), and 5(h)). On the other hand, LPS-induced IL-8 and MCP-1mRNA expression and secretion were significantly reduced in siKRAS transfected trophoblast cells (Figures 5(b), 5(c), 5(f), and 5(g)). Moreover, siKRAS-transfected cells demonstrated significantly reduced IL-6, IL-8, and MCP-1 mRNA expression and secretion when treated with TNF-*α* (Figures 6(a)–6(c), 6(e)–6(g)) or IL-1*β* (Figures 7(a)–7(c), 7(e)–7(g)). GRO-*α* secretion but not mRNA expression (*p* = 0.057) stimulated by TNF-*α* was significantly reduced in siKRAS-transfected cells (Figures 6(d) and 6(h)), whereas IL-1*β*-stimulated GRO-*α* secretion (*p* = 0.054) and expression (*p* = 0.073) were not affected by siKRAS knockdown (Figures 7(d) and 7(h)).

In contrast to the broad suppression of proinflammatory mediators following the loss of K-Ras function in trophoblast cells, loss of H-Ras function in trophoblast cells had limited effect on proinflammatory meditators stimulated by LPS, TNF-*α*, or IL-1*β* (Figures 5–7). siHRAS transfected trophoblast cells showed no effect on IL-6, IL-8, MCP-1, or GRO-*α* mRNA expression or secretion when stimulated by LPS (Figure 5). siHRAS-transfected cells demonstrated a small but significant increase in IL-8 and MCP-1 secretion but not mRNA expression when stimulated with TNF-*α* (Figures 6(b), 6(c), 6(f), and 6(g)). There was no effect on TNF-*α*-induced IL-6 and GRO-*α* expression and secretion with siHRAS knockdown (Figures 6(a), 6(d), 6(e) and 6(h)). In IL-1*β*-stimulated siHRAS-transfected cells, IL-6 and GRO-*α* mRNA expression (Figures 7(a) and 7(d)) was significantly reduced with no effect on IL-6 and GRO-*α* secretion (Figures 7(e) and 7(h)). In contrast, IL-1*β*-induced a small but significant increase in IL-8 secretion in siHRAS-transfected cells, with no effect at the mRNA level (Figures 7(b) and 7(f)). There was no effect on siHRAS on IL-1*β*-induced expression and secretion of MCP-1 (Figures 7(c) and 7(g)).

### 3.5. Effect of siKRAS on ERK Activation in Villous Trophoblast Cells

**Figure 8:**
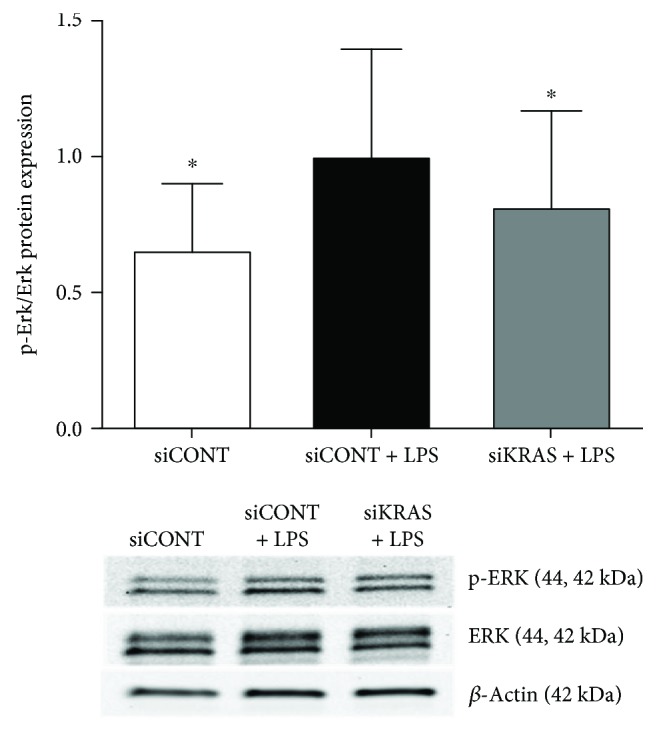
Effect of siKRAS on LPS-induced ERK1/2 activation. Human primary trophoblast cells were transfected with 50 nM siKRAS or 50 nM siCONT for 48 h then treated with 1 *μ*g/ml LPS for an additional 24 h (*n* = 3 patients). Phosphorylated ERK (p-ERK) protein expression was assessed by Western blot and normalised to total ERK. A representative Western blot from 1 patient is shown. The fold change was calculated relative to LPS. ^∗^*p* < 0.05 vs. LPS (repeated measures one-way ANOVA with Fisher's LSD post-hoc test).

**Figure 9:**
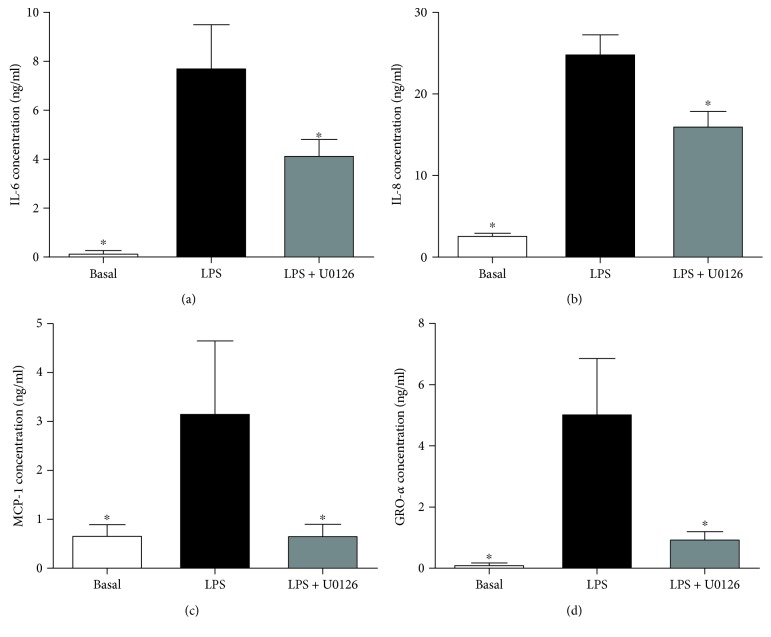
Effect of U0126 on LPS-induced inflammation in human primary trophoblast cells. Human primary trophoblast cells were treated with 1 *μ*g/ml LPS in the presence or absence of 5 *μ*M U0126 for 20 h (*n* = 3 patients). The incubation medium was assayed for concentration of IL-6, IL-8, MCP-1, and GRO-*α* release by ELISA. All data are displayed as mean ± SEM. ^∗^*p* < 0.05 vs. LPS (repeated measures one-way ANOVA with Fisher's LSD post-hoc test).

Ras proteins mediate their downstream effects via the ERK signalling pathway [44]. Following our findings which demonstrate loss of K-Ras function has more of a broader effect in regulating proinflammatory mediators than H-Ras in human placenta, we next sought to investigate the effect of siKRAS on activation of ERK. For these studies, we assessed phosphorylation of ERK (p-ERK) by Western blotting (Figure 8). In siCONT-transfected cells, treatment of trophoblast cells with LPS significantly increased ERK activation as measured by the increase in p-ERK protein expression. siKRAS knockdown resulted in a significant attenuation of LPS-stimulated p-ERK protein expression in trophoblast cells. These findings suggest that K-Ras may regulate LPS-induced proinflammatory cytokines and chemokines via activation of ERK. Thus, to confirm a role for ERK in the regulation of proinflammatory cytokines and chemokines, trophoblast cells were treated in the presence of LPS with or without the specific ERK inhibitor U0126 [22]. As demonstrated in Figure 9, the treatment of trophoblast cells with U0126 significantly suppressed IL-6, IL-8, MCP-1, and GRO-*α* secretion stimulated by LPS.

## 4. Discussion

This is the first study to show that Ras expression and activity is increased in human placenta of obese pregnant women. Loss-of-function studies, using the Ras inhibitor lonafarnib showed a significant reduction in proinflammatory cytokines and chemokines stimulated by LPS, TNF-*α*, or IL-1*β* in placental tissue. K-Ras inhibition significantly reduced the expression and secretion of proinflammatory cytokines and chemokines stimulated by LPS, TNF-*α*, or IL-1*β*. In contrast, there was a limited effect on inflammation following H-Ras siRNA knockdown in trophoblast cells. K-Ras silencing in trophoblast cells resulted in impaired ERK-1/2 activation by LPS. Moreover, we have shown that inhibition of ERK-1/2 activation by its inhibitor U0126 significantly inhibits LPS-induced proinflammatory cytokine and chemokine secretion in trophoblast cells. Taken together, these studies suggest that K-Ras may regulate placental inflammation associated with maternal obesity through the ERK-1/2 signalling pathway.

In obese pregnancies, the expression and secretion of proinflammatory cytokines and chemokines are upregulated in the placenta [14, 15, 17]. Studies in nongestational tissues have reported that chronic inflammation can amplify and prolong Ras activity [45]. In this study, we found increased total Ras (H-Ras, K-Ras, and N-Ras) protein expression and increased K-Ras and H-Ras combined activity in the placenta of obese pregnant women. Therefore, it is possible that this increase in placental Ras expression and activity with obesity could be a consequence of the heightened placental inflammation associated with obese pregnancies. Given that placental Ras expression and activity was increased with maternal obesity, we stimulated primary human trophoblast cells with bacterial endotoxin LPS or with proinflammatory cytokines IL-1*β* or TNF-*α* in order to mimic the inflammatory environment that is associated with obesity [14, 15, 17, 46, 47]. We found that Ras expression and activity was also increased in nonobese placental tissue when stimulated with LPS, TNF-*α*, or IL-1*β*. This finding corroborates other studies in nongestational tissues which have found overexpression of Ras under other inflammatory conditions such as in cancers [48–50], cigarette smoking [51], diabetes, and obesity [52, 53]. In addition to inflammation, apoptotic signals can also activate Ras [54]. Although beyond the scope of the current study, the effects on placental Ras expression following exposure to apoptosis markers such as caspases or PARP (a marker of repair and cell death) warrants further study.

The role of Ras has been extensively studied on its tumorigenic effects in various cancers [29, 49, 51, 52, 55, 56]. Of recent, there is growing evidence to support the influence of K-Ras on inflammation. For example, proinflammatory mediators such as IL-6 and IL-8 are upregulated in response to K-Ras [27, 29]. In a genetic mouse model of K-Ras-mutant pancreatic cancer, oncogenic K-Ras induced the expression of intercellular adhesion molecule-1 (ICAM-1) by pancreatic cells to attract proinflammatory macrophages, leading to increased cytokine storm in the pancreas [56]. Studies in nongestational tissues have reported that Ras induces inflammation through the activation of the proinflammatory transcription factor NF-*κ*B [45]. In this study, we found total Ras protein expression in the placenta was increased with maternal obesity and in response to LPS, TNF-*α*, or IL-1*β* stimulation. To assess the effect of Ras in regulating placental inflammation, placental tissue explants were used to determine the effect of the total Ras inhibition using the farnesyltransferase inhibitor lonafarnib. We found that pretreatment with lonafarnib decreased LPS-, TNF-*α*-, and IL-1*β*-induced inflammation.

Given the increase in total Ras protein expression in the placenta with maternal obesity, we also performed a Ras activity assay which detects both H-Ras and K-Ras activity. We found a significant increase in combined H-Ras and K-Ras activity in obese placenta and in placental tissue exposed to LPS, TNF-*α*, or IL-1*β*. Therefore, we then sought to determine which Ras isoform (H-Ras or K-Ras) was involved in regulating inflammation in human placenta. K-Ras and H-Ras siRNA knockdown was performed in human primary trophoblast cells. In cells transfected with siKRAS, LPS-, TNF-*α*-, or IL-1*β*-stimulated expression of proinflammatory cytokines and chemokines was significantly decreased. Taken together, we have demonstrated the ability of K-Ras to regulate the production of proinflammatory mediators in human placenta in the presence of LPS, TNF-*α*, or IL-1*β*. Given the importance of inflammation in obesity, our studies indicate that K-Ras may play an important role in the mechanisms that contribute to the increased inflammatory state in human placenta associated with maternal obesity.

Studies in nongestational tissues have shown Ras to mediate its downstream effects via activation of ERK [44, 57–59]. In the human placenta, ERK has been shown to regulate LPS-induced secretion of IL-6 and IL-8 [37, 60, 61]. In this study, siRNA knockdown of K-Ras was associated with a significant reduction activation of ERK in the presence of LPS in trophoblast cells. To confirm the role of ERK in LPS-induced inflammation, trophoblast cells were treated with the ERK1/2 inhibitor U0126. Treatment with U0126 resulted in a significant reduction in LPS-stimulated inflammation. Collectively, these findings suggest that K-Ras activates the ERK signalling pathway to mediate inflammation in trophoblast cells.

In contrast to the K-Ras loss-of-function studies, we only have very slight modifications in inflammation induced by H-Ras silencing in trophoblast cells. Studies in nongestational tissues have demonstrated preferential activation of different Ras isoforms between certain cancers. For example, activating mutations in K-Ras are commonly found in colon cancers, hematopoietic malignancies select for N-Ras activation and H-Ras mutations are most common in cancers derived from salivary and pituitary glands [62]. Thus, larger effects observed by K-Ras compared to H-Ras in mediating inflammation in the placenta may be explained by cell type-specific preferential activation of Ras isoforms.

Limitations of this study include total Ras protein expression was measured in lean and obese placenta samples by Western blot. We were unable to optimise Western blotting using specific K-Ras and H-Ras antibodies in placental samples which did not cross-react with other isoforms. Further, K-Ras and H-Ras loss-of-function studies were not assessed in the placenta obtained from obese women; instead, placental tissue and trophoblasts were obtained from the placenta of lean women and were stimulated with LPS TNF-*α* and IL-1*β* to model the inflammatory state of an obese-like environment.

In conclusion, we have demonstrated that total Ras protein expression in the placenta is increased with maternal obesity and when exposed to inflammatory stimuli. Combined H-Ras and K-Ras activity in human placenta was also significantly increased with obesity and inflammation. Lonafarnib, a farnesyltransferase inhibitor of Ras, ameliorated LPS-, TNF-*α*-, and IL-1*β*-stimulated expression and secretion of proinflammatory cytokines and chemokines in human placenta. Additionally, functional studies using K-Ras and H-Ras siRNA knockdown in primary trophoblast cells demonstrated only K-Ras and not H-Ras silencing resulted in a reduction in proinflammatory cytokines and chemokines induced by LPS, TNF-*α*, and IL-1*β*. In trophoblast cells, K-Ras silencing resulted in a significant reduction in ERK-1/2 activation by LPS. We propose that the inflammatory effects mediated by K-Ras in human placenta occur through the ERK-1/2 signalling pathway. Given these findings, K-Ras could act as a potential target for improving both short- and long-term health outcomes of offspring complicated by obese pregnancies. Of promise, lonafarnib is safe for human consumption with clinical trials currently in progress to assess the efficacy of lonafarnib as treatment for a wide range of cancers, chronic hepatitis D, and Hutchinson-Gilford progeria syndrome (premature aging syndrome) [63–65]. However, further studies using i*n vivo* animal models are warranted to determine whether inhibiting K-Ras may be of therapeutic benefit in improving health outcomes of these offspring.

## Figures and Tables

**Table 1:** Patient characteristics.

	Lean cohort (*n* = 15 patients)	Obese cohort (*n* = 15 patients)
Maternal age	31.8 ± 1.1	32.27 ± 0.5
Prepregnancy BMI (kg/m^2^)	23.6 ± 0.6^#^	40.2 ± 2.1^∗^
Delivery BMI (kg/m^2^)	29.0 ± 0.9^#^	42.0 ± 1.9^∗^
Fasting OGTT (mmol/l)	4.3 ± 0.1	4.5 ± 0.1
1 h OGTT (mmol/l)	6.1 ± 0.3	7.2 ± 0.3
2 h OGTT (mmol/l)	5.5 ± 0.2	5.5 ± 0.3
Gestational age (weeks)	38.8 ± 0.2	38.8 ± 0.1
Neonate birth weight (g)	3406 ± 122	3543 ± 139
Fetal gender	8 male; 7 female	5 male; 10 female
Gravida	2.4 ± 0.2	2.8 ± 0.5
Parity	2.0 ± 0.2	2.5 ± 0.4

Values represent mean ± SEM. OGTT: oral glucose tolerance test. ^∗^*p* < 0.05 vs. lean (Mann-Whitney U test).

## Data Availability

The data used to support the findings of this study are included within the article.
